# ^61^Ni synchrotron radiation-based Mössbauer spectroscopy of nickel-based nanoparticles with hexagonal structure

**DOI:** 10.1038/srep20861

**Published:** 2016-02-17

**Authors:** Ryo Masuda, Yasuhiro Kobayashi, Shinji Kitao, Masayuki Kurokuzu, Makina Saito, Yoshitaka Yoda, Takaya Mitsui, Kohei Hosoi, Hirokazu Kobayashi, Hiroshi Kitagawa, Makoto Seto

**Affiliations:** 1 Research Reactor Institute, Kyoto University, Kumatori-cho, Sennan-gun, Osaka 590-0494, Japan; 2 Research and Utilization Division, Japan Synchrotron Radiation Research Institute, 1-1-1 Kouto, Sayo-cho, Sayo-gun, Hyogo 679-5198, Japan; 3 Condensed Matter Science Division, Sector of Nuclear Science Research, Japan Atomic Energy Agency, 1-1-1 Kouto, Sayo-cho, Sayo-gun, Hyogo 679-5148, Japan; 4 Department of Chemistry, Faculty of Science, Kyushu University, 6-10-1, Hakozaki, Fukuoka 812-8581, Japan; 5 Division of Chemistry, Graduate School of Science, Kyoto University, Kitashirakawa-Oiwakecho, Sakyo-ku, Kyoto 606-8502, Japan

## Abstract

We measured the synchrotron-radiation (SR)-based Mössbauer spectra of Ni-based nanoparticles with a hexagonal structure that were synthesised by chemical reduction. To obtain Mössbauer spectra of the nanoparticles without ^61^Ni enrichment, we developed a measurement system for ^61^Ni SR-based Mössbauer absorption spectroscopy without X-ray windows between the ^61^Ni_84_V_16_ standard energy alloy and detector. The counting rate of the ^61^Ni nuclear resonant scattering in the system was enhanced by the detection of internal conversion electrons and the close proximity between the energy standard and the detector. The spectrum measured at 4 K revealed the internal magnetic field of the nanoparticles was 3.4 ± 0.9 T, corresponding to a Ni atomic magnetic moment of 0.3 Bohr magneton. This differs from the value of Ni_3_C and the theoretically predicted value of hexagonal-close-packed (hcp)-Ni and suggested the nanoparticle possessed intermediate carbon content between hcp-Ni and Ni_3_C of approximately 10 atomic % of Ni. The improved ^61^Ni Mössbauer absorption measurement system is also applicable to various Ni materials without ^61^Ni enrichment, such as Ni hydride nanoparticles.

Mössbauer spectroscopy is an efficient method to study the local environment and electronic states of atoms in various compounds, and has been applied to a wide variety of scientific fields. Although most famous and measured element in the Mössbauer spectroscopy is Fe, there are many elements that can be measured. In particular, Ni is an important element in many fields. Therefore, the Mössbauer measurement of Ni is beneficial if it is accessible. ^61^Ni Mössbauer spectroscopy has been used since 1960^1^, although it is often arduous mainly because it requires the preparation of radioactive γ-ray sources. This difficulty is considerably alleviated by using synchrotron radiation (SR) as an alternative source. The high flux density of SR is advantageous because it only requires a small amount of sample. The first ^61^Ni Mössbauer experiment using SR is performed in 2007, when nuclear resonant forward scattering (NFS) of SR was measured for enriched ^61^Ni samples^2^. The NFS is a highly efficient and sensitive method that enables us to measure the hyperfine structure of enriched ^61^Ni metal even at 260 GPa at room temperature^3^. However, analysis of the time spectra of NFS is sometimes difficult for materials with many components, such as inhomogeneous samples^4^. For analysis, energy-domain Mössbauer spectroscopy is more intuitive than the time spectra of NFS. SR-based Mössbauer absorption spectroscopy is an energy-domain method that uses SR^5^,^6^. Furthermore, although it is only a technical point, we can perform this method with any bunch mode of SR except the all continuously filling mode, like the multi-bunch mode in SPring-8 (a SR facility in Japan), uniformly filling mode in European Synchrotron Radiation Facility (ESRF, in France), and 1296 bunches mode in Advanced Photon Source (APS, in USA). Even if the time interval of a bunch mode is so short that it is not desirable of NFS experiments, we can perform SR-based Mössbauer absorption spectroscopy. For example, the “203 bunch” mode with an electron-bunch interval of 23.6 ns in SPring-8 is good for this method, although this interval is only three lifetimes of the ^61^Ni nuclear resonance (lifetime τ = 7.7 ns) and may insufficient for the time spectra of ^61^Ni NFS. Recently, an X-ray windowless measurement system was developed that remarkably enhanced the counting rate in SR-based Mössbauer absorption spectroscopy^7^. In this system, the internal conversion electrons, which were shielded from the detector by X-ray windows, were detected. This system was applied to ^174^Yb and a counting rate five times as high as that of the normal measurement system with X-ray windows was achieved. As a result, the Mössbauer spectrum of natural YbB_12_ without enrichment was successfully observed in 10 h. If we can also observe the Mössbauer spectroscopy of Ni samples without expensive enrichment, this method can be extended to various Ni compounds. We can use compounds which cannot be made stably from small amount of raw materials. Thus, we applied the X-ray windowless measurement system to ^61^Ni SR-based Mössbauer absorption spectroscopy. Moreover, it is applied to the study of hexagonal close-packed nickel (hcp-Ni) nanoparticles.

hcp-Ni is known to be metastable state of Ni metal. It was synthesised as a minor component of a thin Ni film^8^,^9^, and has also been synthesised by chemical reduction^10^. Recently, hcp-Ni nanoparticles have been widely prepared by chemical reduction methods because they are expected to show different properties from those of typical face-centred cubic Ni (fcc-Ni) nanoparticles. For example, hcp-Ni nanoparticles are superior to fcc-Ni ones as a catalyst in aqueous-phase reforming to make hydrogen gas from glycerol, which is a promising method in biomass energy production^11^. However, some researchers suspected that the nanoparticles were contaminated by carbon and formed a structure similar to metastable Ni_3_C, in which the Ni arrangement is the same as that in hcp-Ni^12^,^13^. This ambiguity arose because it is difficult to distinguish hcp-Ni from Ni_3_C by X-ray diffraction (XRD) and normal extended X-ray absorption fine structure experiments^14^. Furthermore, the magnetism of hcp-Ni is still debated. Theoretical calculations predicted ferromagnetism of hcp-Ni with an atomic magnetic moment as large as that of fcc-Ni, approximately 0.6 *μ*_B_ (*μ*_B_: Bohr magneton)^15^,^16^,^17^. However, in most experimental studies, the observed magnetisation of hcp-Ni is at most one-fifth of that of fcc-Ni; for example, 3.66–5.44 emu/g^18^, 6 emu/g^19^, 11.5 emu/g^20^, and 3 emu/g^21^ at 5 T and 5 K *versus* 58.57 emu/g at 0 K (extrapolation) for bulk fcc-Ni^22^ even though their nanoparticles were not saturated. Even if we evaluate the saturation magnetisation of these nanoparticles at 0 K by extrapolation of their experimental values, taking into consideration the temperature dependence of the magnetisation under field cooling (FC) conditions and the magnetisation-external magnetic field hysteresis curve (M-H curve), their saturation magnetisation is probably at most 20 emu/g. Ni_3_C is non-magnetic and thus the low magnetisation obtained for the above nanoparticles also casts doubts on their being composed of hcp-Ni. Conversely, Chiang *et al.*^23^ obtained 70.1 emu/g at 300 K and 5 T for an intermediate phase in the decomposition of Ni_3_C. In addition, surface and finite-size effects can result in the magnetic properties of nanoparticles being different from those of the bulk material. Magnetic impurities can also affect the magnetic properties of nanoparticles. Thus, there are many factors to consider when investigating hcp-Ni nanoparticles. Local information around Ni atom should be helpful to elucidate further details of hcp-Ni nanoparticles. Mössbauer spectroscopy is an effective tool to the study the local state of nanoparticles of Fe^24^,^25^ and also used for the study of Ni nanoparticles^26^,^27^,^28^. Thus, we applied ^61^Ni SR-based Mössbauer spectroscopy to the hcp-Ni nanoparticles. In fact, the hyperfine magnetic field (HF) of ^61^Ni nuclide is better for the comparison with theoretically calculated atomic magnetic moment than the magnetisation from the mass of nanoparticles. In this article, we report the detail of the X-ray windowless system for ^61^Ni SR-based Mössbauer spectroscopy and the application to non-enriched Ni nanoparticles with hexagonal structure synthesised by chemical reduction.

## Methods

**Figure 1:**
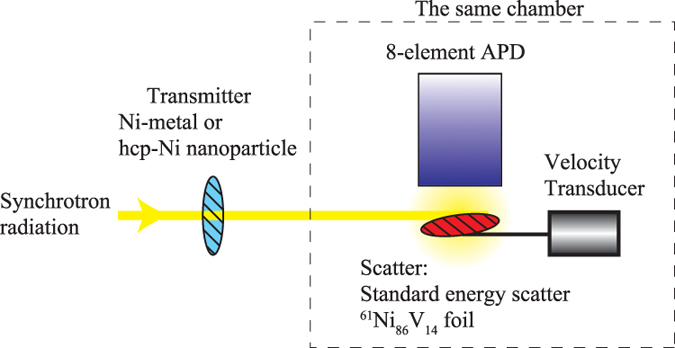
Schematic diagram of the experimental setup for ^61^Ni SR-based Mössbauer absorption spectroscopy. The line with the arrow indicates the SR path.

SR-based Mössbauer absorption spectroscopy was performed at the undulator beamlines (BL09XU: public beamline for nuclear resonant scattering (NRS) experiments, BL11XU: Contract beamline of Japan Atomic Energy Agency) of SPring-8. The experimental setup is shown in Fig. 1. The operating mode of the electron-storage ring was the “203 bunch” mode. The SR from the undulator was monochromatised to the energy of the first nuclear excited state of ^61^Ni, *E*_res_ = 67.4 keV, by using Si (333) high heat load monochromator (HHLM). Behind the HHLM, the low-energy noise filter blocked the low-energy X-rays by Si (111) reflection of HHLM. The filter consisted of two Si (111) monochromators in channel-cut like arrangement at BL09XU, and a 0.3 mm Cu plate at BL11XU. The former can perfectly reduce the low energy X-rays but the adjustment of the optical system is a little difficult. The latter is very simple, although 0.36% of the low energy X-rays pass through it, while 73% of the 67.4 keV X-rays pass. Here, the size of SR was typically 1.5 mm (horizontal) × 0.5 mm (vertical). Then the 67.4-keV SR were transmitted through the sample (“transmitter” in Fig. 1), which was Ni metal in the feasibility study or the Ni nanoparticles described later. The former was non-enriched polycrystalline Ni metal (99% purity) formed into a plate with dimensions of 5 × 5 × 1 mm^3^; this thickness corresponded to 0.9 g(Ni)/cm^2^. The latter was the nanoparticle powder. The nanoparticle powder was formed into a pellet with a 5-mm diameter with a thickness of 0.5 g(Ni)/cm^2^. Downstream of the transmitter, the SR was scattered by ^61^Ni-enriched Ni_86_V_14_ (^61^Ni enrichment 86.2%) polycrystalline foil (“standard energy scatterer” in Fig. 1). The thickness of the scatterer was 3.1 ± 0.3 μm (Ni_86_V_14_), and it was inclined 30° from the horizontal plane. The relative nuclear resonance energy of the scatterer to that of the transmitter was controlled by a conventional velocity transducer connected to the scatterer, because the velocity of the scatterer modulated its resonance energy through the Doppler effect of light. The temperature of the transmitters ranged from 4 to 60 K and that of the scatterer was typically 30 K. The emission from the scatterer was detected using an eight-element Si avalanche photodiode (APD) detector. Each element of the detector had a surface area of 3 × 5 mm and its depletion layer was approximately 150 μm thick. In this system, the detector was installed in the same vacuum chamber as the scatterer, and there was no X-ray window between the detector and scatterer. Thus, the detector counted not only the elastically scattered γ-rays and fluorescent X-rays following the internal conversion process, but also the internal conversion electrons. From this point we call this system as the X-ray windowless system. The details of this system are reported in ref. 7. The NRS was typically counted in a time window between 3 and 20 ns after the SR pulse because NRS by ^61^Ni is emitted with a delay (typically the lifetime of ^61^Ni), while the electronic scattering was scattered promptly. Using this system, the intensity of the NRS from the scatterer was observed as a function of the velocity of the scatterer. When the resonance energy of the scatterer at a velocity corresponded to the resonance energy of the transmitter, the transmitter absorbed the SR at that energy through nuclear resonance, and thus the detected intensity decreased. Conversely, when the resonance energy of the scatterer at another velocity did not correspond to the resonance energy of the transmitter, the transmitter did not absorb the SR at the scatterer’s resonance energy through nuclear resonance and thus the detected intensity did not decrease. In this way, we obtained energy-domain Mössbauer absorption spectra. Therefore, the counting rate in this system depended on the photoelectronic absorption by the transmitter and the NRS intensity from the standard energy scatterer, although the absorption depth of the Mössbauer spectrum depends on the nuclear resonance absorption by the transmitter. From this point of view, the arrangement around the scatterer and the detector was improved in the X-ray windowless system, compared with those in the normal system with X-ray windows between the two.

**Figure 2:**
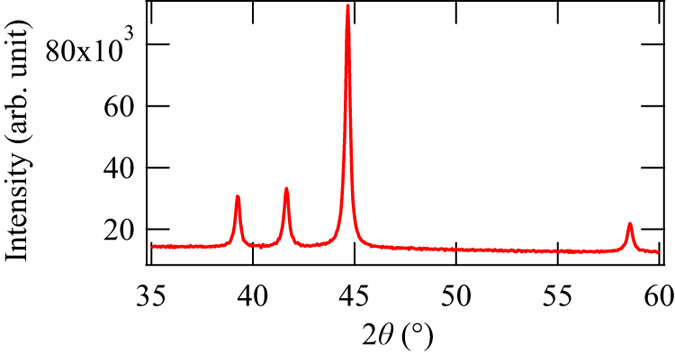
XRD pattern of the hcp nanoparticles at room temperature.

**Figure 3:**
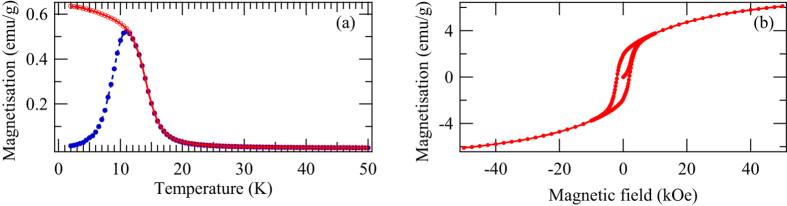
Magnetisation measurements, (**a**) Temperature dependence of the magnetisation with an external field of 50 Oe under FC and ZFC conditions. The red solid line with open circles is under the FC condition and the blue dashed line with filled circles is under the ZFC condition. (**b**) M-H curve measured at 2 K. The circles are the data points and the lines are visual guides in both (**a**) and (**b**).

The nanoparticles used in this experiment were synthesised by chemical reduction method. 0.045 mmol of NiCl_2_·6H_2_O, 4.7 mmol of NaOH, and 23.5 mmol of polyvinylpyrrolidone (PVP) were dissolved in triethyleneglycol at 140 °C. Then a reducing agent N_2_H_4_·H_2_O was added and the mixture was heated to 250 °C. It was maintained at the temperature in 20 min and then cooled down rapidly with ice bath. The Ni sample was washed with acetone, diethyl ether and water by repeating the centrifugation and re-dispersion several times. PVP was used to prevent the nanoparticles from aggregating, and thus the nanoparticles were coated with PVP. The XRD pattern of this nanoparticle powder was observed by the Bruker D8 Advance powder diffractometer with Cu Kα sources. The XRD pattern of the nanoparticles (Fig. 2) was consistent with hcp structure with lattice constants of *a* = 0.2655 nm and *c* = 0.4349 nm. The crystallite size was typically 40 nm according to the Scherrer equation. The temperature dependence of the magnetisation of this nanoparticle powder with FC and zero-field cooling (ZFC) conditions (Fig. 3a) and the M-H curve at 2 K (Fig. 3b) were observed by the Quantum Design magnetic property measurement system. From these magnetisation measurements, the Curie temperature of the nanoparticles was 15 K and its magnetisation was 6 emu/g under 5 T at 2 K, although the M-H curve of the nanoparticles was not saturated even under 5 T. In addition, the blocking temperature for magnetisation measurement was 11 K. These values were similar to the other hcp-Ni nanoparticles in the literatures^18^,^19^,^20^,^21^.

## Results and Discussion

The typical counting rate of NRS emitted from the scatterer Ni-V alloy in our system was 1.3 × 10^2^ counts per seconds (cps) with a constant noise of 4 cps. This counting rate is more than twice high counting rate of 60 cps, that is the rate from the same Ni-V alloy obtained by a four-element fast scintillation detector for high-energy X-rays using the normal system with two X-ray windows between the scatterer and the detector^6^: one X-ray window was at the chamber for the scatterer and the other was at the package for the detector. The reason for this enhancement is the detection efficiency and solid angle from the scatterer. Regarding the detection efficiency, the main detected signal in the X-ray windowless system was internal conversion electrons and its efficiency was almost 100%, while the main signal detected in the normal system with a scintillation detector was the directly scattered 67.4-keV X-rays, and its efficiency was 29%. Although the probability of internal conversion is only 13.9% of the direct scattering of 67.4 keV X-rays in the ^61^Ni nuclear resonance, the electron detection must contribute to the high counting rate. Meanwhile, the solid angle of the detection area to the scattering point is larger in the X-ray windowless system than that in the system with X-ray windows because the distance between the scatterer and surface of the detector elements is smaller. In fact, this distance was 4 mm in the X-ray windowless measurement system. The large solid angle caused by this nearness also contributes to the high counting rate. As a result, the spectra of the non-enriched Ni metal and hcp-Ni nanoparticle powders were measured within 20 h by the X-ray windowless system, while the measuring time of the ^61^Ni-enriched superconducting sample with a Ni content of 3% was 52 h by the normal system in ref. 6. We note that further enhancement of counting rates will be achieved by the combination of this system and a detector for high-energy X-rays to detect the directly scattered X-rays, because the probability of direct scattering is more than seven times higher than that of internal conversion in ^61^Ni. The detector for high energy X-rays continues to be improved^29^. Another approach to improve the efficiency is to shorten the distance between the scatterer and detector. In principle, this distance can be as small as 2 mm but this closeness also introduces strong thermal radiation from the detector to the scatterer. Therefore, to further shorten the distance between the scatterer and the detector, we need to carefully arrange radiation shields.

**Figure 4:**
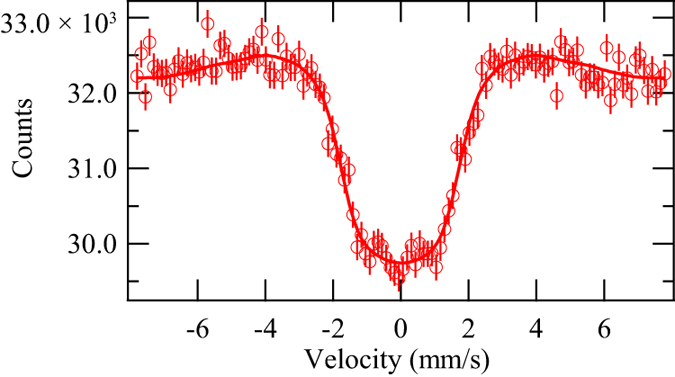
^61^Ni SR-based Mössbauer spectrum of Ni metal at 20 K. The open circles show the raw data and the solid line shows the fitting. The error bars of the raw data are statistical errors.

The SR-based Mössbauer spectrum of natural Ni metal at 20 K was measured as a feasibility study. The spectrum is shown in Fig. 4. In the analysis of the ^61^Ni spectra described below, the equations in ref. 5 based on ref. 30 were used with the following parameters from ref. 31: the nuclear quadrupole moment of the ground state *Q*_g_=+0.162 barn, and that of the first excited state *Q*_e_ = −0.20 barn, the nuclear magnetic moment of the ground state *μ*_g_ = −0.75002 *μ*_N_ (*μ*_N_: nuclear magneton), and that of the first excited state *μ*_e_ = +0.480 *μ*_N_. Because the nuclear spin moment of the ^61^Ni ground state is 3/2 and that of the excited state is 5/2, a ^61^Ni Mössbauer spectrum with a hyperfine magnetic field (HF) consists of 12 transitions, while a ^61^Ni Mössbauer spectra without HF and quadrupole splitting (QS) consists of 1 degenerated transition^32^. With these conditions, the obtained hyperfine parameters using a least-square fitting were as follows: the isomer shift (IS) relative to Ni_84_V_16_ at 30 K (including second-order Doppler shift) was −0.02 ± 0.04 mm/s, the quadrupole splitting (QS) was 0.0 ± 0.1 mm/s, and the HF was 7.7 ± 0.2 T. Here, the IS and HF obtained by the traditional Mössbauer spectroscopy using a radioactive ^61^Co source in Ni_86_V_14_ at 4.2 K by Love and co-workers were −0.000 ± 0.003 mm/s and 7.6 ± 0.1 T, respectively^33^. The QS was little that they could not see the effect of the QS in various Ni compounds except NiF_2_, in ref. 33. Considering the Curie temperature of Ni metal, 627 K, the atomic magnetic moment of Ni should be actually saturated at low temperatures and thus the HF of Ni metal obtained at 20 K in the SR-based Mössbauer absorption spectrum and that obtained at 4.2 K is consider to be consistent in the experimental error. For the IS, we should evaluate the second order Doppler shift as the temperature dependent factor. However, the difference of the second Doppler shift at 4.2 K and that at 20 K is evaluated to be less than 0.001 mm/s in the framework of Debye model using the Debye temperature of 380 K^34^. Furthermore, the ionicity of Ni metal should not differ in both temperatures. Therefore, the IS from the SR-based Mössbauer spectrum and that by Love is also consistent. The zero QS from SR-based Mössbauer absorption is also consistent with the condition in the literature. The obtained HF is also consistent with the value obtained from the NFS of SR of 7.4 ± 0.2 T at 3.2 K^2^. This result confirms that ^61^Ni SR-based Mössbauer absorption spectroscopy with the X-ray windowless system can be used to extract the hyperfine structure of various Ni compounds. It is noted that the values of IS and QS in ^61^Ni Mössbauer spectra are small and not as simple to use as those in ^57^Fe spectra, as discussed in refs 32 and 33. Therefore, we henceforth concentrate on the HF.

**Figure 5:**
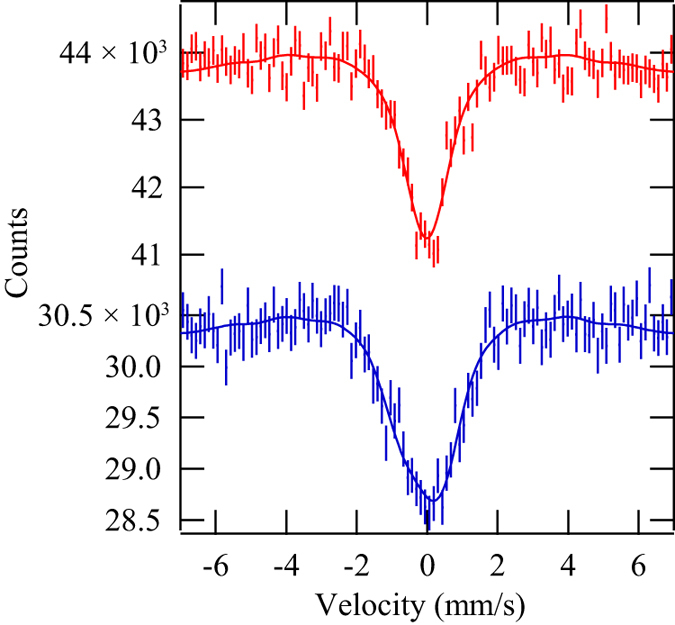
^61^Ni SR-based Mössbauer absorption spectra of hexagonal Ni nanoparticles. The upper spectrum was observed at 60 K and the lower at 4 K. The measurement time for each spectrum was 17.5 h. The dots show the raw data and the solid lines show the fittings. The error bars of the raw data are statistical errors.

^61^Ni SR-based Mössbauer absorption spectra of the Ni nanoparticles with hexagonal structure are presented in Fig. 5. The spectra were measured at 4 and 60 K, which are respectively below and above the Curie temperature of the nanoparticles of 15 K. The Mössbauer spectrum measured at 4 K is wider than that obtained at 60 K because of the HF of the nanoparticles. These spectra are consistent with the nanoparticles containing a single component. The IS, QS, and HF were −0.01 ± 0.03 mm/s, 0.00 ± 0.02 mm/s, and 0.0 ± 0.2 T at 60 K, respectively. Those were 0.0 ± 0.1 mm/s, −0.3 ± 0.8 mm/s, and 3.4 ± 0.9 T at 4 K, respectively. Because components at 7.7 T corresponding to fcc-Ni and 10 T corresponding to nickel oxide (NiO) were not found at 4 K, the content of fcc-Ni and oxidisation of surface Ni in the nanoparticles is below the statistical error of these spectra (approximately 10%). The spectrum measured at 4 K did not contain a non-magnetic component, and therefore the content of non-magnetic Ni_3_C is also below the statistical error. Therefore, our nanoparticles were not a mixture of simply ferromagnetic hcp-Ni and non-magnetic Ni_3_C, which cannot be distinguished by simple XRD. Also, because the theoretically predicted ferromagnetic hcp-Ni should show HF similar to the fcc-Ni, it is not included in the nanoparticles. However, the observed HF of nanoparticles was sometimes lower than that of bulk, because of the collective excitation effect. Thus, it is not still perfectly excluded that the nanoparticles are composed of a mixture of fcc-Ni, NiO, and/or theoretical hcp-Ni. The collective excitation effect is caused by the thermal fluctuation of the atomic magnetic moment in nanoparticles and lowers the observed HF of nuclei at temperatures below the blocking temperature of superparamagnetism. Although the blocking temperature for ^61^Ni Mössbauer spectroscopy has not been directly observed, we can estimate it from the blocking temperature for M-H curve measurements. Here, the blocking temperature *T*_Bn_ (n = χ for magnetisation measurements and n = M for ^61^Ni Mössbauer spectroscopy) satisfies the Néel–Arrhenius equation for the magnetic fluctuation of the nanoparticles; *t*_mn_ = *τ*(*T*_Bn_) = *τ*_0_exp(*K*_u_*V*/*k*_B_*T*_Bn_), where *t*_mn_ (n = χ or M) is the typical measurement time of the method, *τ* is the Néel relaxation time of thermal fluctuation, *τ*_0_ is the attempt time, which is typically 10^−9^–10^−13^ s, *k*_B_ is the Boltzmann constant, *K*_u_ is the magnetic anisotropy constant of the nanoparticle, and *V* is the volume of a nanoparticle. *t*_mχ_ is typically 10^2^ s and *t*_mM_ is 10^−9^ s; *t*_mM_ depends on the nuclear Larmor precession and the critical relaxation time for the nuclear transition from a excited state to a ground state is written as *τ*_cr_ =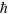 /|(*μ*_e_*m*_e_−*μ*_g_*m*_g_)*B*_hf_|^32^; here, 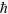 is the Planck constant over 2π, *m*_j_ are the principle axis components of the nuclear spin of nuclear state j (j = g for ground state and j = e for excited state), and *B*_hf_ is the HF. From the Néel–Arrhenius equations for the M-H curve measurement and Mössbauer spectroscopy, *T*_BM_/*T*_Bχ_ = (ln*t*_mχ_−ln *τ*_0_)/(ln*t*_mM_ − ln*τ*_0_)). Using *T*_Bχ_ = 11 K obtained from the magnetisation measurements, we obtain *T*_BM_ > 40 K. Thus, the conditions for collective magnetic excitation are satisfied at 4 K. The observed HF *B*_hf_ob_ at temperature *T* caused by collective magnetic excitation is expressed as *B*_hf_ob_ = *B*_hf_(1 − *k*_B_*T*/2*K*_u_*V*) when *T* is sufficiently lower than *T*_BM_^24^,^34^. We can also evaluate the anisotropy energy *K*_u_*V* from the Néel–Arrhenius equation for the M-H curve as *K*_u_*V* = *k*_B_*T*_Bχ_ (ln*t*_mχ_ − lnτ_0_). Thus, *B*_hf_ = *B*_hf_ob_/{1 − *T*/[2(ln*t*_mχ_ − ln*τ*_0_)*T*_Bχ_]} < *B*_hf_ob_(4 K)/0.9919 = 3.5 ± 0.9 T. This value does not agree with those of fcc-Ni, NiO and theoretical hcp-Ni, and thus the possibilities of the nanoparticles composed of fcc-Ni, NiO, and/or theoretical hcp-Ni can be completely excluded. One likely possibility is the presence of nickel carbide with less carbon than Ni_3_C. In fact, carbon may be supplied by the surrounding PVP or triethyleneglycol used as a solvent during the nanoparticle synthesis. As for the atomic magnetic moment of Ni in NiC_*x*_, Schaefer *et al.*^13^ experimentally observed a gradual decrease of magnetisation of NiC_*x*_ with increasing carbon content, and Fang *et al.*^17^ calculated the decrease of the atomic magnetic moment of Ni in NiC_*x*_ as *x* increases. If we consider that NiC_*x*_ is ferromagnetic, as predicted by Fang *et al.*, our nanoparticles might be NiC_*x*_ with *x* < 0.33. In fact, the HF of ^61^Ni is a probe of the atomic magnetic moment of Ni. According to the 3.4 T HF, the estimated atomic magnetic moment of Ni is 0.3 *μ*_B_, assuming that the proportionality of the atomic magnetic moment of Ni to HF of ^61^Ni in these nanoparticles is the same as those in bulk fcc-Ni, which has a 7.7 T HF and atomic magnetic moment of Ni of 0.6*μ*_B_. We can evaluate the carbon concentration from this atomic magnetic moment of Ni of 0.3 *μ*_B_ according to Fang and colleagues, who calculated the atomic magnetic moment of NiC_*x*_ for the crystal structure of P6_3_22, which the carbon site in is different from that in the actual Ni_3_C structure of R
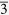
c. Nevertheless, their calculations are sufficiently relevant to allow rough estimation of the carbon concentration in our nanoparticles. From the atomic magnetic moment of 0.3 *μ*_B_, the chemical composition of our nanoparticles is evaluated to be NiC_0.1_.

It is also useful to compare the HF determined from ^61^Ni Mössbauer spectroscopy and the magnetisation measured from the corresponding M-H curve. If our nanoparticles possess simple ferromagnetic order, their 0.3 *μ*_B_ atomic magnetic moment, evaluated from the HF obtained by Mössbauer spectroscopy, corresponds to magnetisation about 30 emu/g. In contrast, the observed magnetisation was 6 emu/g at 2 K under 5 T. If we evaluate the saturation magnetisation at 2 K from the shape of the M-H curve by extrapolation, it was at most 8 emu/g. Thus, the saturation magnetisation of our nanoparticles at 4 K was less than 8 emu/g. The difference between 30 emu/g and 8 emu/g implies these nanoparticles did not possess simple ferromagnetic order. One likely possibility for this difference is a surface effect. The magnetic structure of a surface region can differ from that of the core region; for instance, canting of the magnetic moment^25^. In fact, Du *et al.*^36^ found that the saturation magnetisation in the M-H curve of their fcc-Ni nanoparticles was lower than that of bulk fcc-Ni, and they attributed this to a surface effect. For Fe nanoparticles, ^57^Fe Mössbauer spectroscopy has been used to distinguish the component in the core of the nanoparticle and the one in its surface. However, the resolution of our ^61^Ni spectra may be insufficient to separate the core and surface components which have small difference of IS, QS, and/or HF and to see the small distribution of HF, reflecting the slightly different Ni sites, although they were discussed by ^57^Fe Mössbauer spectra. In fact, the spectrum at 60 K showed the resolution of our spectra; the full width at half maximum (FWHM) was 1.2 mm/s, when the spectrum was evaluated using a Lorentzian function. However, this resolution can be improved at the expense of counting rate by setting the appropriate time window^37^. In fact, about 2/3 of the 2*Γ* (*Γ*: natural line width of nuclear resonance) was obtained in the time window after the 2.4 times of the life time of ^174^Yb in ^174^Yb SR-based Mössbauer absorption spectroscopy. If this condition is applied to ^61^Ni Mössbauer absorption spectroscopy, the FWHM would be decreased to about 0.5 mm/s. With this resolution, further evaluation of this surface effect would be possible. This width-narrowing effect is also important for the use of IS, whose change by Ni ionicity is typically less than 0.1 mm/s^33^,^38^. Considering these viewpoints, further enhancement of the counting rate is still important.

As described above, ^61^Ni Mössbauer spectroscopy is an effective method complementary to structural measurements like XRD and many bulk measurements like M-H curve. ^61^Ni Mössbauer spectroscopy allowed us to identify impurities, the number of constituents, and state of each constituent. In fact, this evaluation method can also be applied to nitrides and hydrides. The absorption of these atoms also induces a change in atomic magnetic moment and the dependence of the moment of Ni on the concentration of N and H has been discussed by Fang *et al.*^17^ and Vergas *et al.*^39^ respectively. Thus, the amount of N or H in Ni nanoparticles can be determined in the same way as carbon. ^61^Ni Mössbauer spectroscopy is a useful technique to study these materials. Moreover, our system can also tolerate any sample provided the 67-keV highly penetrating X-rays transmit through it. Thus, ^61^Ni Mössbauer spectroscopy can be performed under extreme conditions using instruments like a high pressure cell or gas chamber. Furthermore, we can focus the SR on the sample cell using the Kirkpatrick-Baez optics, which have already been applied in the ^61^Ni NFS and realized a beam size of 15 μm × 15 μm in ESRF^3^. For measurements under special conditions, it is also advantageous that the evaluation process of the HF from the Mössbauer spectrum does not require exact values of parameters of the sample like weight. This is convenient for the study of nanoparticles because it is sometimes difficult to estimate their exact sample weight, especially when coated by protective materials. In addition, when an unexpected reaction occurs, estimation of their weight becomes even more difficult. Fortunately, even in such a case, the HF is not affected by ambiguity of the sample weight.

We also add a remark on the applicability of this system to nuclides other than ^61^Ni. Because the internal conversion coefficient *α* of ^61^Ni is 0.139, which is low compared with those of many other Mössbauer-active nuclides like ^174^Yb (*α* = 9.43), the detection of internal conversion electrons appears inefficient. However, our results show that the X-ray windowless system is also efficient to enhance the counting rate of SR-based Mössbauer absorption spectroscopy using nuclides with low *α*, such as ^40^K (*α* = 0.298), ^73^Ge (third excited state, *α *= 0.8), and ^155^Gd (*α *= 0.43). Recently, ^40^K Mössbauer spectroscopy was applied to potassium nanoclusters in a porous crystal of sodalite, and used to confirm the antiferromagnetic ordering of potassium^40^.

In summary, we observed ^61^Ni SR-based Mössbauer spectra of hcp-Ni nanoparticles using an X-ray windowless system. Although the nanoparticles were not enriched, the enhanced counting rates achieved by this system compensated for the low density of ^61^Ni. The Mössbauer spectra of hcp-Ni revealed that the contents of impurity phases fcc-Ni and NiO were below the detectable limit, and the nanoparticles consisted of a single material. An HF of 3.4 ± 0.9 T was obtained at 4 K, and this HF can be understood by the presence of nickel carbide. From the HF, the Ni atomic magnetic moment was roughly estimated to be 0.3*μ*_B_ and the carbon content of the Ni nanoparticles was estimated to be NiC_0.1_ in the framework of ferromagnetism. Comparison of the Mössbauer spectrum and the magnetisation measurements indicated some local magnetic structure in the nanoparticles which may arise from surface effects. This method may be extended to nanoparticles of Ni compounds including hydrides and nitrides, as well as to various conditions like high pressure and gas atmosphere.

## Additional Information

**How to cite this article**: Masuda, R. *et al.*^61^Ni synchrotron radiation-based Mössbauer spectroscopy of nickel-based nanoparticles with hexagonal structure. *Sci. Rep.* **6**, 20861; doi: 10.1038/srep20861 (2016).
