# Erratum: ^61^Ni synchrotron radiation-based Mössbauer spectroscopy of nickel-based nanoparticles with hexagonal structure

**DOI:** 10.1038/srep27053

**Published:** 2016-06-02

**Authors:** Ryo Masuda, Yasuhiro Kobayashi, Shinji Kitao, Masayuki Kurokuzu, Makina Saito, Yoshitaka Yoda, Takaya Mitsui, Kohei Hosoi, Hirokazu Kobayashi, Hiroshi Kitagawa, Makoto Seto

Scientific Reports 6: Article number: 20861;10.1038/srep20861 Published online: 02 17 2016; Updated: 06 02 2016

In this Article, there is an error in the Author Contribution section.

“R.M. and M.Seto designed this study. K.H., H.Kobayashi and H.Kitagawa synthesised the hexagonal Ni nanoparticles and measured XRD, M-H curves, and magnetisation under ZFC and FC conditions. R.M., M.Seto, Y.K., S.K., M.K., Y.Y. and T.M. performed the SR-based Mössbauer absorption spectroscopy. R.M., M.Seto, Y.K., S.K., M.K. and M.Saito analysed the data. R.M. and M.Saito mainly co-wrote the most of manuscript and all authors contributed to writing the manuscript”.

should read:

“R.M. and M.Seto designed this study. K.H., H.Kobayashi and H.Kitagawa synthesised the hexagonal Ni nanoparticles and measured XRD, M-H curves, and magnetisation under ZFC and FC conditions. R.M., M.Seto, Y.K., S.K., M.K., Y.Y. and T.M. performed the SR-based Mössbauer absorption spectroscopy. R.M., M.Seto, Y.K., S.K., M.K. and M.Saito analysed the data. R.M. and M.Seto mainly co-wrote the most of manuscript and all authors contributed to writing the manuscript”.

